# TRPV1 dysfunction in cystinosis patients harboring the homozygous 57 kb deletion

**DOI:** 10.1038/srep35395

**Published:** 2016-10-13

**Authors:** L. Buntinx, T. Voets, B. Morlion, L. Vangeel, M. Janssen, E. Cornelissen, J. Vriens, J. de Hoon, E. Levtchenko

**Affiliations:** 1 Center for Clinical Pharmacology, Department of Pharmaceutical and Pharmacological Sciences, KULeuven, Herestraat 49, 3000 Leuven, Belgium; 2 Department of Cellular and Molecular Medicine, KULeuven, Herestraat 49, 3000 Leuven, Belgium; 3 Center for algology and pain management, Department of Cardiovascular Sciences, KULeuven, Weligerveld 1, 3212 Pellenberg, Belgium; 4 Department of internal medicine, Radboud UMC Nijmegen, Geert Grooteplein-Zuid 22, 6525 GA Nijmegen, The Netherlands; 5 Department of Pediatric Nephrology, Radboud UMC Nijmegen, Geert Grooteplein-Zuid 22, 6525 GA Nijmegen, The Netherlands; 6 Department of Development and Regeneration, KULeuven, Herestraat 49, 3000 Leuven, Belgium.

## Abstract

Cystinosis is a rare autosomal recessive disorder characterized by lysosomal cystine accumulation due to loss of function of the lysosomal cystine transporter (CTNS). The most common mutation in cystinosis patients of Northern Europe consists of a 57-kb deletion. This deletion not only inactivates the *CTNS* gene but also extends into the non-coding region upstream of the start codon of the *TRPV1* gene, encoding the capsaicin- and heat-sensitive ion channel TRPV1. To evaluate the consequences of the 57-kb deletion on functional TRPV1 expression, we compared thermal, mechanical and chemical sensitivity of cystinosis patients with matched healthy controls. Whereas patients heterozygous for the 57-kb deletion showed normal sensory responses, homozygous subjects exhibited a 60% reduction in vasodilation and pain evoked by capsaicin, as well as an increase in heat detection threshold. Responses to cold, mechanical stimuli or cinnamaldehyde, an agonist of the related nociceptor channel TRPA1, were unaltered. We conclude that cystinosis patients homozygous for the 57-kb deletion exhibit a strong reduction of TRPV1 function, leading to sensory deficiencies akin to the phenotype of TRPV1-deficient mice. These deficits may account for the reported sensory alterations and thermoregulatory deficits in these patients, and provide a paradigm for life-long TRPV1 deficiency in humans.

Cystinosis (OMIM 219800) is rare autosomal recessive disorder (incidence of 1 case per 100,000 to 200,000 live births), characterized by lysosomal cystine accumulation causing renal Fanconi syndrome, growth retardation, vomiting, periods of dehydration, constipation, and sometimes rickets. Lysosomal cystine accumulation is due to an impairment of the lysosomal membrane exodus of cystine, resulting from mutations in the cystinosin gene (*CTNS*) on chromosome 17p13.2^1^,^2^. More than the half of the alleles of cystinosis patients in Northern Europe are affected by a 57-kb deletion^3^. This deletion not only inactivates the *CTNS* gene but also removes the entire *CARKL* (carbohydrate kinase like) gene, which encodes a sedoheptulose kinase, and extends into the non-coding region of the *TRPV1* gene. Freed *et al.*^4^ reported reduced TRPV1 mRNA expression in peripheral blood mononuclear cells from cystinosis patients homozygous for the 57-kb deletion. Whereas the deletion of the *CARKL* gene is known to result in increased concentrations of sedoheptulose in tissues, plasma and urine of patients^5^, the potential (patho)physiological consequences of the 57-kb deletion on *TRPV1* gene function are unknown.

TRPV1 (transient receptor potential vanilloid 1), a member of the TRP superfamily of cation channels, is highly expressed in a subset of primary sensory neurons, more specifically in terminals of small- to medium-diameter nociceptors, such as peptidergic and nonpeptidergic C fibers, as well as some Aδ fibers^6^. TRPV1 is directly activated by heat, protons and by a variety of chemical ligands that evoke sensations of heat and/or pain, of which the best known example is capsaicin, the pungent compound from hot chili peppers^7^. In the peripheral nervous system, TRPV1 activation is not only associated with pain, but also with neurogenic inflammation^8^. Indeed, activation of TRPV1 at sensory nerve endings causes local release of neuropeptides, including calcitonin gene-related peptide (CGRP), which results in vasodilation and edema formation^8^. Based on observations in *Trpv1*^−/−^ mice, which lack sensitivity to capsaicin and show strongly reduced thermal hyperalgesia after inflammation and injury, much effort has been devoted to the development of TRPV1 antagonists as a potential novel analgesic drugs^6^,^9^,^10^,^11^. Several classes of TRPV1 antagonists have been developed, which showed efficacy in various preclinical and clinical trials. However, hyperthermia and impaired heat perception were frequent unwanted on-target side effects, raising doubts about the safety of long-term TRPV1 antagonism in clinical practice^12^,^13^,^14^,^15^.

Interestingly, several cases of cystinosis patients with disturbances in thermoregulation, such as recurrent bouts of fever and heat intolerance, as well as craving for hot and spicy food have been reported, which could potentially be related to altered functional expression of TRPV1^16^,^17^,^18^. To evaluate this possibility, we examined heat and capsaicin sensitivity in cystinosis patients.

## Results

To evaluate the potential influence of the 57-kb deletion on TRPV1 function, we compared chemical, mechanical and thermal sensitivity of cystinosis patients that were either homozygous or heterozygous for the deletion, with matched healthy controls. Detailed information on mutations in individual patients is provided in Table 1.

### Demographics

Comparison of demographics between these three groups is provided in Table 2. No significant differences were found in age, blood pressure, heart rate or BMI between the three groups.

All cystinosis patients took cysteamine to treat the cystine accumulation. No difference was found in cysteamine dose (g/m^2^/day) between patients with homo- or heterozygous 57 kb deletion. Both in the homozygous and the heterozygous groups, about half of the patients had a kidney graft and received immunosuppressive treatment. There were no significant differences between the two patient groups with respect to the use of anti-hypertensive drugs. All patients without kidney graft had renal Fanconi syndrome characterized by polyuria, proteinuria and urinary loss of several substances like glucose, amino acids, uric acid, phosphate and bicarbonate. All transplanted patients had a stable renal function. Body core temperature was normal in all patients. Comorbidities at the moment of the study are listed in Table 1.

### Reduced capsaicin sensitivity in homozygous patients

**Figure 1:**
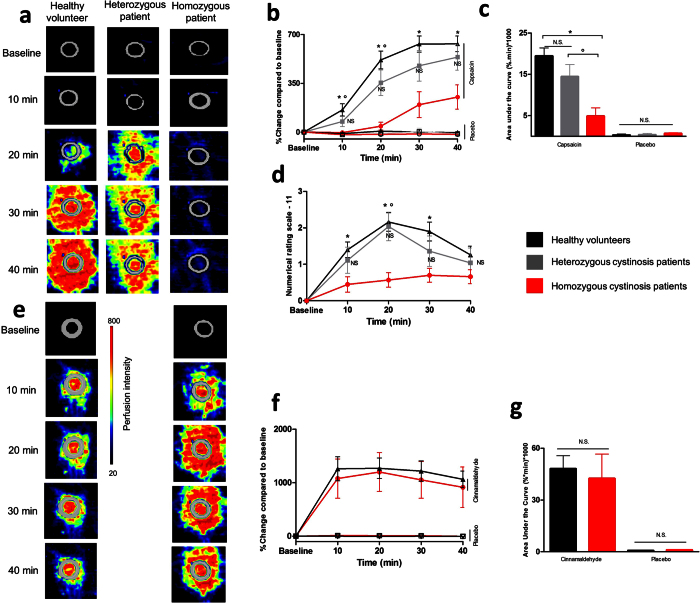
Sensitivity of cystinosis patients to topical application of capsaicin or cinnamaldehyde. (**a**) LDI images showing responses to topical capsaicin application in a healthy volunteer and in a heterozygous and a homozygous cystinosis patient. (**b**) Mean time course of the DBF expressed as % change from baseline in response to capsaicin or placebo (=vehicle), in healthy volunteers (n = 25), heterozygous patients (n = 14) and homozygous patients (n = 11). (**c**) Mean area under the curve for the data in (**b**). (**d**) Capsaicin induced pain, expressed in NRS-11 over time for the subjects in (**b**). (**e**) LDI images showing responses to topical cinnamaldehyde application in a healthy volunteer and in a homozygous cystinosis patient. (**f**) Mean time course of the DBF expressed as % change from baseline in response to cinnamaldehyde or vehicle, in healthy volunteers (n = 5) and homozygous patients (n = 5). (**g**) Mean area under the curve for the data in (**f**). *p < 0.05 between homozygous patients and healthy volunteers. °p < 0.05 between homozygous patients and heterozygous patients (Kruskall-Wallis with post-hoc Dunn’s (capsaicin) or Mann-Whitney-U (cinnamaldehyde)) N.S.: Non-significance. Data are presented as mean ± SEM.

To investigate capsaicin sensitivity, a capsaicin-containing solution or vehicle was applied in small rubber rings on the volar surface of the left arm, and Laser Doppler scanning was performed at 10-min interval to measure capsaicin-induced changes in dermal blood flow (DBF). In addition, the Numerical Rating Scale (NRS)-11 pain scale was used to quantify capsaicin-induced pain at these time points. In healthy volunteers, capsaicin induced a large increase in DBF during the first 40 minutes of application, as well as a clear pain response that peaked at 20 minutes. In contrast, capsaicin responses were blunted in cystinosis patients homozygous for the 57-kb deletion: the onset of the DBF response was delayed, and both the objective peak DBF response as well as the subjective pain levels were reduced by more than 60% compared to healthy volunteers. Notably, in heterozygous patients capsaicin-induced changes in DBF and pain responses were not significantly different from the healthy controls (Fig. 1a–d). The same results were found when excluding patients who received a kidney graft, which suggests that DBF responses were not noticeably influenced by immune suppressive treatment used after renal transplantation (Suppl. Fig. 1). These results indicate that reduced capsaicin sensitivity is not a general property of cystinosis patients but only of patients with bi-allelic deletion of the 57-kb genomic fragment.

Capsaicin-induced increases in DBF are the result of the release of vasoactive peptides such as CGRP from nerve terminals in the skin^8^. To exclude the possibility that the reduced DBF response in the homozygous patients was secondary to impaired neuropeptide release from terminals and/or reduced responsiveness of the vasculature to these neuropeptides, we developed an assay in which we evaluated changes in DBF evoked by topical cinnamaldehyde. Cinnamaldehyde is an agonist of TRPA1, a nociceptor ion channel that is highly co-expressed with TRPV1 in nerve terminals, and whose activation causes CGRP release in animal models^19^,^20^,^21^,^22^. We found that cinnamaldehyde evokes robust increases in DBF, and these increases were virtually identical in the homozygous patients and the healthy volunteers (Fig. 1e–g). In addition, all three groups showed a similar response to the placebo (=vehicle) solutions used for both capsaicin and cinnamaldehyde (Fig. 1b,c,f,g).

### Deficit in heat sensitivity in homozygous patients

**Figure 2:**
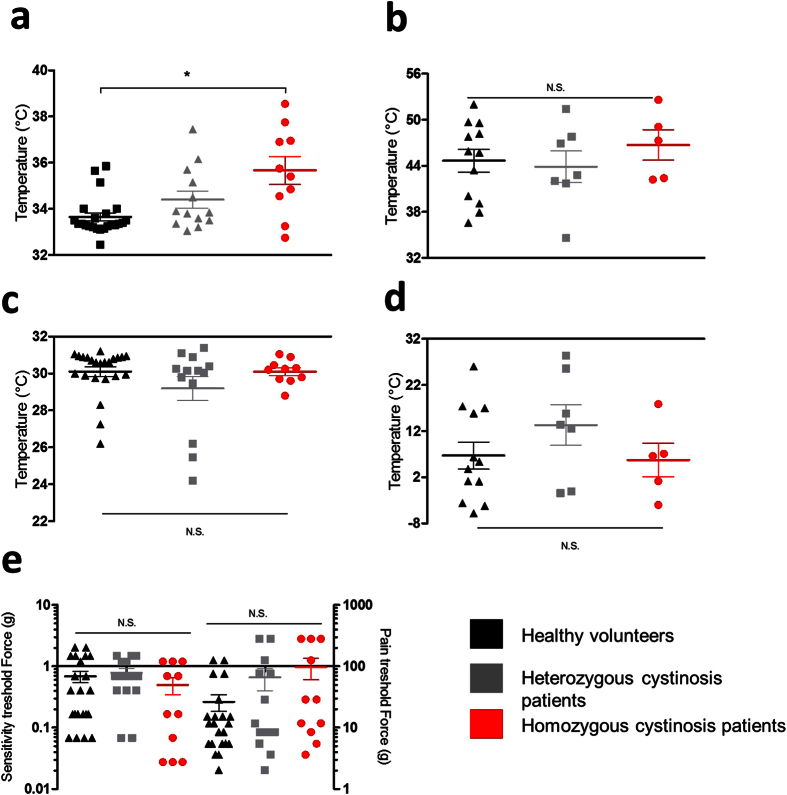
Sensitivity of cystinosis patients to thermal and mechanical stimuli. (**a–d**): Heat detection thresholds (**a**), heat pain thresholds (**b**), cold detection thresholds (**c**) and cold pain thresholds (**d**) in healthy volunteers, heterozygous patients and homozygous patients. (**e**) Detection thresholds and pain thresholds for mechanical stimuli applied via von Frey hairs in healthy volunteers, heterozygous patients and homozygous patients. *p < 0.05 between homozygous patients and healthy volunteers (Kruskall-Wallis with post-hoc Dunn’s or One-way ANOVA with post-hoc Bonferroni). N.S.: Non-significance. Data are presented as mean ± SEM.

Since TRPV1 is a heat-activated channel, and has been implicated in heat sensing and thermoregulation in animal models and humans, we compared sensitivity to heating and cooling in the homo- and heterozygous patients to that in healthy volunteers. Analogous to earlier work in patients with persistent pain and healthy controls^23^, we evaluated both the temperatures at which the subjects first detected a change in temperature (heat/cold detection threshold), as well as the temperatures at which the warming or cooling evoked pain (heat/cold pain thresholds). Homozygous patients showed a significantly higher heat detection threshold (35.7 ± 0.6 degrees) compared to healthy volunteers (34.3 ± 0.6 degrees; Fig. 2a). Heat pain thresholds were not significantly different between the groups, although it should be noted that, in line with the literature^23^, this parameter showed fairly large interindividual variability, thereby reducing the power of the comparison. We also did not detect any significant differences between the three groups with respect to their cold detection or cold pain thresholds (Fig. 2c,d). To evaluate whether the 57-kb deletion in cystinosis has a more general effect on somatosensory sensitivity, we also assayed skin mechanosensitivity using von Frey hairs. These experiments revealed no noticeable differences in detection threshold or pain threshold between the three groups, indicating that mechanosensitivity is not affected in cystinosis (Fig. 2e).

### Correlation with subjective reporting

There are several, mostly anecdotal reports in the literature that cystinosis patients have a more frequent preference for spicy food or disturbances in thermoregulation than healthy subjects, but whether this is related to TRPV1 dysfunction is unknown. To address whether the objectively determined alterations in capsaicin sensitivity and thermosensation in the homozygous patients correlated with their subjective experiences in daily life, we performed a short structured interview in both hetero- and homozygous patients who were asked (1) whether they had a preference for spicy food, and (2) whether they had the feeling that the regulation of their body temperature was disturbed. Interestingly, 67% (6 out of 9) of the homozygous patients reported a strong preference for spicy food, compared to only 25% of the heterozygous patients (3 out of 12; p = 0.039). Likewise, 78% (7 out of 9) of the homozygous patients reported subjective thermosensory/thermoregulatory problems, such as hot flashes, difficulties in adapting to changes in temperature or difficulties in sensing temperature differences, compared to only 25% of the heterozygous patients (3 out of 12; p = 0.014).

## Discussion

In this study, we provide evidence that homozygous deletion of the 57-kb fragment in cystinosis patients results in functional impairment of the TRPV1 receptor, as evidenced by a strongly reduced responsiveness to the selective TRPV1 activator capsaicin. The reduced capsaicin-induced vasodilation and pain responses in the homozygous patients recapitulate the reduced pain response and vasodilation reported both in TRPV1-deficient mice and in humans treated with TRPV1 antagonists^9^,^24^,^25^. Importantly, the reduced response to capsaicin was not the consequence of an overall deficit in vasodilation or sensitivity to chemical irritants, as homozygous patients showed unaltered vasodilatory responses to cinnamaldehyde, an agonist of the related nociceptor channel TRPA1.

In addition to the strong deficiency in capsaicin sensitivity, we also measured milder alterations in heat sensitivity in the homozygous patients. In particular, these patients had a significantly higher threshold for detecting a heating stimulus. Their average threshold for heat pain was 46.7 ± 2.0 degrees compared to the threshold of healthy volunteers 44.7 ± 1.5 degrees, but no significant difference was found (p = 0.6), possibly due to the limited size of the patients group that we have access to, combined with the established inter-individual differences in heat pain thresholds^26^. It should be noted here that even in mice the effects of TRPV1 deletion on acute noxious heat sensing are, at best, very mild. Indeed, in the initial descriptions of *Trpv1*^*−/−*^ mice, Caterina *et al.* reported longer withdrawal latencies in the hot plate test, but only at temperatures exceeding 52 degrees, whereas Davis *et al.* reported unaltered nocifencive behavior in the hot plate assay^9^,^27^. Similarly, in human volunteers treated with the TRPV1 antagonist SB-705498, noxious heat thresholds were increased by not more than 1–2 degrees^24^. There is accumulating evidence for the existence of additional noxious heat sensors in the sensory system, including other TRP channels (TRPM3, TRPV3, TRPV4) and the heat-sensitive calcium-activated chloride channel ANO1 (TMEM16A), which may (at least partially) compensate for the loss of TRPV1 function in detecting acute heat stimuli in mice and humans^26^,^28^,^29^,^30^.

Since heterozygous cystinosis patients, who all lacked functional cystinosin due to the 57-kb deletion on one allele and another *CTNS* mutation on the other allele, did not exhibit any difference in capsaicin responses or heat sensitivity compared to healthy subjects, we can exclude that reduced capsaicin sensitivity represents a general effect of the disease cystinosis (or of the drugs taken to manage the disease and/or kidney transplant) on sensory processes or vascular responses. Instead, it suggests that the sensory deficit represents a direct consequence of homozygous 57-kb deletion on functional TRPV1 expression. The phenotype observed in our heterozygous patients is also in line with *Trpv1*^*+/−*^ mice, which show no difference in their responses to capsaicin and temperature compared to the wild-type mice^27^.

The depletion of the sedoheptulose kinase CARKL is reportedly associated with increased immune response^27^, however, we consider it unlikely that the reduced vascular response to capsaicin is related to the deletion of the *CARKL* gene in the homozygous patients. The TRPV1 dependence of the sensory deficit is further supported by our finding that neither vasodilatation in response to the TRPA1 agonist cinnamaldehyde, nor responses to cold or mechanical stimuli were affected in the homozygous patients.

In spite of the reduced TRPV1 functionality, the homozygous patients generally presented with a normal core body temperature. This may seem at odds with studies showing that healthy volunteers dosed with first generation TRPV1 antagonists exhibited mild to severe hyperthermia^13^,^14^,^31^,^32^. However, it was also shown that these hyperthermic response disappeared upon repeated dosing^12^. Moreover, recent evidence suggests that modality-specific antagonists, which inhibit TRPV1 activity induced by heat or capsaicin but not by protons, do not markedly affect body temperature^33^. Finally, the *Trpv1*^*−/−*^ mice also showed a normal core body temperature. Taken together, our and others’ data indicate that pharmacological or genetic reduction of TRPV1 activity in humans does not necessarily result in permanent alternations of body core temperature. Moreover, considering the role of TRPV1 in pathological pain conditions, the non-coding region upstream of the TRPV1 gene could be a possible target to reduce functional TRPV1 for therapeutic reasons. However, further evidence is required to validate whether homozygous patients have altered inflammatory pain experiences.

There have been anecdotal reports of cystinosis patients preferring hot and spicy food, or exhibiting disturbances in thermoregulation^16^,^17^,^18^, but whether this correlated with altered TRPV1 functionality remained unknown. To address this issue, we asked both heterozygous and homozygous cystinosis patients whether they preferred spicy food or felt that they experienced problems with thermosensation or thermoregulation. Interestingly, 67% of the homozygous patients reported a preference for spicy food and 78% reported subjective thermosensory or thermoregulatory deficits, such as difficulties in sensing small temperature differences or the occurrence of hot flashes. In comparison, only 25% of the heterozygote patients made similar reports. We speculate that these self-reported daily life experiences in the homozygous patients are a direct consequence of the reduced TRPV1 functionality in their sensory neurons, which impairs their ability to respond to challenges of their thermoregulatory system, and renders them less sensitive to capsaicin-rich spicy food. Considering that objective parameters such as blood pressure and body core temperature in the homozygous patients were normal and indistinguishable from that in the heterozygous patients, we estimate that the physiological consequences of the reduced TRPV1 functionality are overall mild. More systematic studies are required to confirm these observations.

Our study did not unravel the exact genomic mechanism whereby the 57-kb deletion affects functional expression of TRPV1. A possible explanation is that the 57-kb deletion influences the interaction of transcription factors that regulate TRPV1 expression^4^. In line herewith, it has been reported that TRPV1 mRNA levels are reduced in peripheral blood mononuclear cells from homozygous patients^4^. It should be noted, however, that functional expression of TRPV1 in non-sensory cells such as blood cells is low, and that transcriptional regulation of TRPV1 expression is likely to be different in sensory neurons^28^,^34^,^35^. Unfortunately, since it is not possible to sample sensory neurons from living patients, we had no means to measure TRPV1 expression at the mRNA level in these cells. Nevertheless, our current findings provide the first direct evidence that the genomic deletion results in actual functional deficits in the sensory system that can be directly linked to the known properties of TRPV1.

In conclusion, we demonstrate that cystinosis patients homozygous for the 57-kb deletion exhibit a significant reduction in capsaicin-induced dermal vasodilatation and pain, and thus provide a paradigm for life-long TRPV1 dysfunction in humans.

## Methods

### Patients and healthy volunteers

Cystinosis patients were recruited from the University Hospitals Leuven, Belgium and the University Medical Center Radboud Nijmegen, the Netherlands. The diagnosis of cystinosis was based on the clinical presentation, measurement of elevated white blood cell cystine levels and confirmed by a molecular analysis of the *CTNS* gene. Healthy volunteers were recruited via the Center for Clinical Pharmacology, University Hospitals Leuven. The study was approved by the Ethics Committees of both hospitals (BE: registration nr.: ML8725, approved at 16 Nov 2012; NL: registration nr.: 2013/122/NL42764.091.12, approved at 14 May 2013) and conducted according to the declaration of Helsinki and International Guidelines on Clinical Trials of Medicinal Products (ICH/GCP Topic E6–July 1996) (ClinicalTrials.gov identifier: NCT02533076). All participants (or their parents when <18y) provided written informed consent prior to their participation in the study.

Male and female participants were eligible if they were >8 years and did not have any abnormalities of the forearm skin or allergies to capsicum plants. All participants refrained from alcohol or caffeine for at least 12 hours and fasted for 3 hours before the study visit. Healthy volunteers had to be good health and took no medication. Patients and healthy volunteers were matched head-to-head based on age and sex.

### Mutational analysis of the *CTNS* gene

57 kb deletion was detected by PCR analysis as described by Heil *et al.*^3^. Direct Sanger sequencing of the *CTNS* coding exons and exon-intron boundaries was performed to detect other mutations.

### Laser Doppler imaging & capsaicin application

Three O-shaped rubber rings (Quad Ring BS011 NBR 70 Shore A; Polymax Ltd) were placed on the volar site of the left arm of the subject. After 30 minutes of acclimatization, baseline DBF was measured and 10 minutes hereafter, capsaicin was applied in the two proximal rings (referred to as I1 and I2) and placebo in the distal ring (I3).

Capsaicin powder and Trans-cinnamaldehyde oil were obtained from Sigma-Aldrich N.V. (Bornem, Belgium). Capsaicin was dissolved in a 3:3:4 mixture of ethanol 100%, Tween-20 and distilled water to obtain a 300 μg/20 μl (for subjects <18 years old) or 1000 μg/20 μl (>18 years old) concentration. The 1000 μg/20 μl dose of capsaicin was used in adults as previous studies reported that this dose produces the most robust response^36^. In children (<18y old), 300 μg/20 μl was used based on pilot experiments showing that 300 μg capsaicin already induced a strong pain response, and that the DBF response was as robust as the 1000 μg dose in adults (data not shown). Trans-cinnamaldehyde oil was dissolved in a 3:3:1 mixture of ethanol 100%, Tween-20 and distilled water to obtain a 10% concentration. The placebo solution corresponded to the same mixtures without the active substances. The 300- or 1000-μg dose capsaicin or the 10% cinnamaldehyde were always applied in the two proximal rings, placebo in the distal ring. The application of capsaicin/trans-cinnamaldehyde started on I1. The laser Doppler scans were performed 10 minutes after application of capsaicin/transcinnamaldehyde, using the Laser Doppler perfusion imager (Periscan PIM III, Perimed AB, Sweden). During 40 minutes, the 3 rings on the left arm were scanned every 10 minutes. This capsaicin DBF model has been used extensively in clinical studies, developed and validated by Van der Schueren *et al.*^36^. Sample size calculations were also based on Van der Schueren *et al.*^36^.

### Numerical rating scale (NRS-11)

The pain level induced by the capsaicin was determined by asking the subject to score his pain with the numerical rating scale (NRS-11). Zero stood for ‘no pain at all’ and 10 ‘the worst pain you could imagine’. The pain level was determined at baseline (i.e. after 30 minutes of acclimatization) and 10, 20, 30 and 40 minutes after capsaicin application.

### Von Frey test

A set of 17 monofilaments (Somedic, Sweden), marked 3 to 19 with different diameters (0.12–1 mm), was applied in ascending and descending sequence on the skin of the forearm on the area of capsaicin application with appropriate force (0.026–110 g) for it to obtain a U-shape. Each filament was applied 3 times to the skin with intervals of 1 second. The subjects were asked to report how many times they felt the application and how many times the application was painful.

### Advanced thermal stimulation

The sensitivity of the skin for temperature, hot and cold, was determined using the advanced thermal stimulator (ATS) (Pathway ATS, Medoc, Israel). The device uses a thermode (contact area of 27 mm diameter), with a baseline temperature of 32 °C, that was attached to the thenar eminence at the base of the thumb. The method used was the method of limits as described by Rolke *et al.*^37^,^38^ and Malmström *et al.*^23^. First, the detection threshold for cold was determined by cooling down the thermode 4 times (10 seconds inter-stimuli intervals) at a rate of 1 °C/second, starting from the baseline temperature of 32 °C. The subject was asked to click immediately on a computer mouse when the decrease in temperature was noticed. This was done in a similar way to find the detection threshold for heat, now by for warming up the thermode at a rate of 1 °C/second. Next, cold and heat pain thresholds were determined. Here, the subject was asked to click on the computer mouse when the temperature of the thermode became ‘painful’. This was repeated 3 times (10 seconds inter-stimuli intervals) for both heating and cooling down of the thermode with a rate of 1 °C/second, starting from the baseline temperature of 32 °C.

### Patients’ interview

A short, exploratory structured interview was performed by the investigator, during which the following two questions were asked (translated from Dutch): (1) “Do you have a preference for spicy food?”, and (2) “Do you have the feeling that the regulation of your body temperature is disturbed?”

### Statistical analyses

Values are expressed as means ± standard error of the mean.

When comparing between 3 independent groups, one-way ANOVA was performed for continuous normally distributed variables (temperature induced pain, demographics) and Kruskall-Wallis test was performed for continuous non-normally distributed (CIDBF, AUC, temperature perception) or discrete variables (NRS-11, Von Frey scores). Shapiro-Wilk test was used to check for normality. Levene’s test was used to check equality of variances. Post-hoc Bonferroni or Dunn’s multiple comparison test was used to correct for multiple testing. When comparing two independent groups with small sample size (cinnamaldehyde induced DBF and AUC), the Mann-Whitney test was used. For analysis of the data from the exploratory, structured interview, Barnard’s exact test was used. Statistical significance was taken at the 5% level (p value < 0.05). Statistical software of Statistica^®^ and Graphpath Prism^®^ was used.

## Additional Information

**How to cite this article**: Buntinx, L. *et al.* TRPV1 dysfunction in cystinosis patients harboring the homozygous 57 kb deletion. *Sci. Rep.* **6**, 35395; doi: 10.1038/srep35395 (2016).

## Supplementary Material

Supplementary Information

## Figures and Tables

**Table 1:** Patients’ demographics, spicy food preference and thermoregulation.

Pt	Sex	Birth year	Genotype	Renal status	Spicy food preference	Feeling of disturbed thermo- regulation^a^	Comorbidities^b^
1°	M	1996	57 kb del + c.926dup	FS	+	−	Growth hormone deficiency
2	F	1975	57 kb del + c.926dup	Tx	−	−	Growth retardation, kidney stones
3	M	1989	57 kb del + undetected mutation^c^	Tx	+	−	None
4°	F	1990	57 kb del + c.198_218del21	Tx	−	−	None
5	F	2003	57 kb del + c.198_218del	FS	−	−	None
6	M	1990	57 kb del + c.696dup	Tx	-	+	None
7	F	1987	57 kb del + del exon5	Tx	−	+	None
8	M	1987	57 kb del + c.1015G > A	Tx	+	−	None
9°	F	1995	57 kb del + c.665A > G	FS	ND	ND	None
10°	F	1995	57 kb del + c.665A > G	FS	ND	ND	None
11°	M	1986	57 kb del + del exon5	Tx	−	+	None
12*°	F	2004	57 kb del + c.926 dup	FS	−	−	None
13°	F	2005	57 kb del + c.926dup	FS	−	−	Genua valga
14°	M	1997	57 kb del + c.198_218del21	Tx	−	−	Lumbal scoliosis
15°	M	1995	Hom 57 kb del	FS	ND	ND	None
16	F	1993	Hom 57 kb del	FS	ND	ND	None
17^×^	M	1987	Hom 57 kb del	Tx	+	−	Acne vulgaris
18^×^	F	1983	Hom 57 kb del	Tx	+	+	Ophthalmologic migraine
19^×^	M	2000	Hom 57 kb del	FS	+	+	None
20^×^	F	1997	Hom 57 kb del	Tx	−	+	None
21^×^	M	1979	Hom 57 kb del	Tx	+	+	Growth retardation
22*°	M	1999	Hom 57 kb del	FS	−	+	Pes plani and genua valga
23°	M	1990	Hom 57 kb del	FS	+	−	Buritis elbow
24°	F	1992	Hom 57 kb del	Tx	+	+	Candida foot nails
25°	M	1999	Hom 57 kb del	FS	−	+	Genua valga

FS: Fanconi Syndrome. TX: Renal transplantation. ND: no data. del = deletion, dup = duplication. *These patients (and matching healthy volunteers) did not participate in the temperature detection test (Fig. 2a,c). °These patients (and matching healthy volunteers) did not participate in the temperature pain threshold test (Fig. 2b,d). ^×^These patients (and matching healthy volunteers) participated in the cinnamaldehyde test (Fig. 1e–g). ^a^Spice food preference and thermoregulation based on self-reporting of the patients during exploratory, structured interview. ^b^Comorbidities at time of measurements. ^c^Although the mutation on the second allele in this patient couldn’t be detected, the diagnosis of cystinosis was based on elevated white blood cell cystine level and the detection of corneal cystine crystals.

**Table 2:** Summary demographics of all 3 groups.

Parameter^a^	Healthy volunteers (n = 25)	Heterozygous patients (n = 14)	Homozygous patients (n = 11)
Age - yrs.	20.7 ± 1.2	20.3 ± 2.0	21.2 ± 2.0
Male sex – no (%)	13 (52)	6 (42)	7 (63)
BMI - kg/m^2^	20.7 ± 1.4	20.5 ± 1.0	19.4 ± 1.0
Kidney graft – no (%)	NA	57%	54%
BP			
Systolic BP - mmHg	120 ± 2	121 ± 3	120 ± 4
Diastolic BP- mmHg	67 ± 2	72 ± 3	72 ± 3
HR –bpm	69 ± 2	74 ± 3	77±4
Smoker–no (%)	0 (0)	1 (7)	1 (9)
Medication use – no (%)			
Anti-hypertensives	NA	9 (78)	9 (81)
Immunosuppressive drugs	NA	6 (57)	6 (54)
Cysteamine (mg/m^2^/day)	NA	1.19 ± 0.13	1.34 ± 0.11
Body temperature (°C)^b^	NA	36.4 ± 0.2	36.5 ± 0.4

^a^Plus-minus values are means ± SEM. There were no significant differences between the groups (p > 0.05).

^b^Body temperature measurements were based on temperature measurements recorded in the medical files of the patients during the year of the study. NA = not applicable.
