# Supplementary material for: TRPV1 dysfunction in cystinosis patients harboring the homozygous 57 kb deletion
Source: Sci Rep. 2016 Oct 13;6:35395. doi: 10.1038/srep35395 (PMC5062165; doi:10.1038/srep35395)
Supplement: Supplementary Information [file srep35395-s1.pdf]

TRPV1 dysfunction in cystinosis patients harboring the homozygous 57kb deletion

L. Buntinx[1*], T. Voets[2], B. Morlion[3], L. Vangeel[2], M. Janssen[4], E. Cornelissen[5], J. Vriens[6], J. de Hoon[1,°], E. Levtchenko[6,°]

[1]Center for Clinical Pharmacology, Department of Pharmaceutical and Pharmacological Sciences, KULeuven, [2]Department of Cellular and Molecular Medicine, KULeuven, [3]Center for algology and pain management, Department of Cardiovascular Sciences, KULeuven, [4]Department of internal medicine, Radboud UMC Nijmegen, [5]Department of Pediatric Nephrology, Radboud UMC Nijmegen, [6]Department of Development and Regeneration, KULeuven.

* Correspondence to linde.buntinx@uzleuven.be

°These authors contributed equally to this work

Non-randomized clinical study approved by the Medical Ethics Committees of the University Hospitals Leuven, Belgium (registration nr.: ML8725, approved at 16 Nov 2012) and the University Medical Center Radboud Nijmegen, the Netherlands (registration nr.: 2013/122; NL42764.091.12, approved at 14 May 2013) and conducted according to the declaration of Helsinki and International Guidelines on Clinical Trials of Medicinal Products (ICH/GCP Topic E6 – July 1996) (ClinicalTrials.gov identifier: NCT02533076).

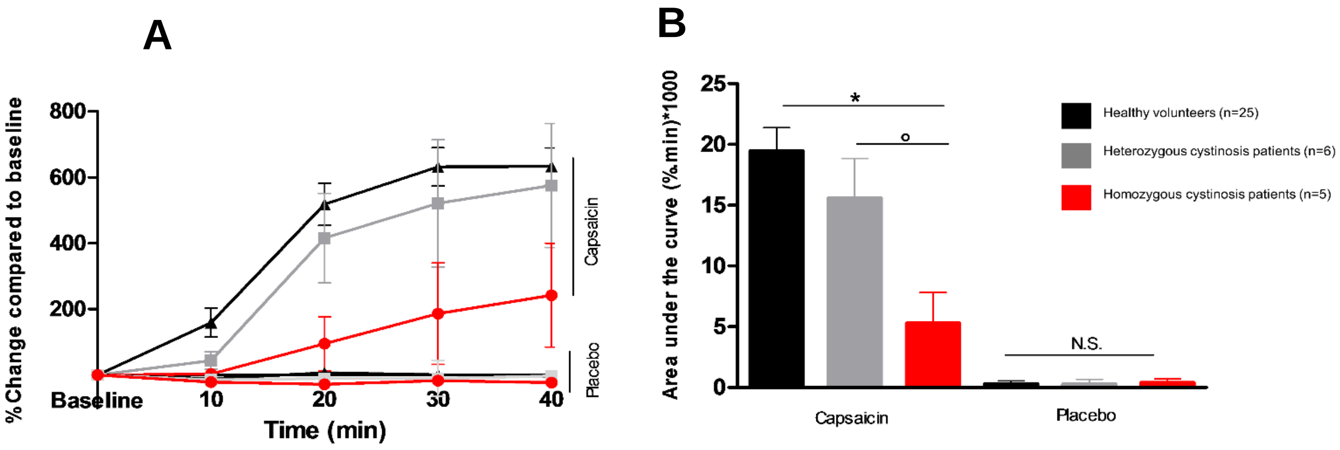

Supplementary figure 1: Sensitivity of cystinosis patients without renal transplantation to topical application of capsaicin.

To exclude the influence of renal transplantation and/or immunosuppressive drugs, transplanted patients are excluded from this analysis.

(A) Mean time course of the DBF expressed as % change from baseline in response to capsaicin or vehicle, in healthy volunteers, heterozygous patients and homozygous patients.

(B) Mean area under the curve for the data in (A).

*: $p<0.05$ between homozygous patients and healthy volunteers.°: $p<0.05$ between homozygous patients and heterozygous patients (Kruskall-Wallis with post-hoc Dunn’s). N.S.: Non-significance. Data are presented as mean ± SEM.
